# The Hospital World

**Published:** 1911-07-29

**Authors:** 


### THE HOSPITAL WORLD.

### The New Governors of Leicester Infirmary.

The following lady and gentlemen have been elected Life Governors of the Leicester Infirmary: Miss S. Heron and the Rev. James Went, M.A. (headmistress and headmaster of the Wyggeston Schools), whose scholars have maintained two cots in the Children's Hospital; Mr. Samuel Folwell, who had contributed £105; and Mr. Oswald Stoll (managing director of the Leicester Palace Theatre), and Mr. Arthur Baum, the Hon. Treasurer of the local movement, for organising an annual matinée at the theatre.

### The League of Roses at the Great Northern Central.

Her Highness Princess Victoria of Schleswig-Holstein visited the Great Northern Central Hospital and distributed badges of merit to members of the League of Roses, an organisation exclusively of women, of which her Highness is president. Miss Roby, the chairman of the League of Roses, said that the movement had grown in a marvellous way from a little seed, the object being to raise £1,000 annually in support of a women's surgical ward in the hospital. Islington and the neighbourhood surrounding the hospital having been marked out in four divisions, each with a separate organising committee, many hundreds of members had joined the various divisions, the result of the first half of the year being that upwards of £450 had been collected in half-crowns, shillings, sixpences, and even pence. The distinctive mark of the association is a rose, the various colours red, white, pink, and yellow representing the four divisions. After the ceremony her Highness visited the wards of the hospital and expressed herself much pleased with their appearance.

